# Nurses’ Perceptions of Facilitating Advance Care Planning Conversations in the Emergency Department

**DOI:** 10.1089/pmr.2020.0116

**Published:** 2021-03-15

**Authors:** Mohammad Adrian Hasdianda, Tamryn F. Gray, Josephine Lo Bello, Brittany Ballaron, Natasha A. Egorova, Donna L. Berry, Kei Ouchi

**Affiliations:** 1 Department of Emergency Medicine, Brigham and Women’s Hospital, Boston, Massachusetts, USA.; 2 Department of Emergency Medicine, Harvard Medical School, Boston, Massachusetts, USA.; 3 Department of Psychosocial Oncology and Palliative Care, Dana-Farber Cancer Institute, Boston, Massachusetts, USA.; 4 Phyllis F. Cantor Center for Research in Nursing and Patient Care Services, Dana-Farber Cancer Institute, Boston, Massachusetts, USA.; 5 Department of Medicine, Division of Palliative Medicine, Brigham and Women’s Hospital, Boston, Massachusetts, USA.; 6 Department of Medicine, Harvard Medical School, Boston, Massachusetts, USA.; 7 University of North Carolina at Chapel Hill School of Medicine, Chapel Hill, North Carolina, USA.; 8 Serious Illness Care Program, Ariadne Labs, Boston, Massachusetts, USA.

**Keywords:** advance care planning, ED GOAL, emergency medicine, motivational interview, nurse-led

## Abstract

**Background:**

Nurses are well positioned to initiate advance care planning (ACP) conversations because of their unique strength in communication and central patient-facing role in the interdisciplinary team. Nurse-led ACP conversations have demonstrated promising results in settings outside of the emergency department (ED). Understanding ED nurses’ perspectives regarding ACP conversations is needed before implementing similar practices in the ED.

**Objective:**

To explore ED nurses’ perception of facilitating ACP conversations.

**Design:**

We conducted a cross-sectional survey to assess ED nurses’ perceptions of facilitating ACP conversations in the ED.

**Setting:**

ED nurses at one academic hospital and one community hospital located within the northeastern region of the United States.

**Results:**

Seventy-seven (53.1%) out of 145 eligible ED nurses completed the survey. All participants perceived ACP conversations in the ED as at least somewhat important. Forty (51.9%) felt somewhat comfortable in facilitating these conversations. The majority of participants (77.9%) agreed that a specially trained nurse consultation model might be helpful in the ED. We found a correlation between total clinical experience and interest in facilitating ACP conversations in the ED (*p* = 0.045).

**Conclusion:**

ED nurses are well positioned to help patients clarify their goals-of-care and end-of-life care preferences. They perceived ACP conversations to be important and felt comfortable to facilitate them in the ED. Additional studies are needed to empirically test its implementation.

## Introduction

In older adults with serious life-limiting illnesses, advance care planning (ACP) conversations can lead to well-informed shared decision making and improved quality of life.^1^ Such conversations are associated with lower rates of in-hospital death, less aggressive medical care at the end of life, earlier hospice referrals, increased peacefulness, decreased stress and anxiety among caregivers, and a 56% greater likelihood to have end-of-life wishes known and followed.^1–8^ Yet only 37% of seriously ill older adults have ACP conversations with their clinicians, on average 33 days before death.^9^ During the last six months of life, 75% of older adults visit the emergency department (ED),^10^ signaling a more rapid decline in their illness trajectories.^11–13^ At this point, a strong impetus exists to motivate seriously ill older adults to seek ACP conversations.^14^

Our research team developed *ED GOAL*, a six-minute motivational interview to empower older adults in the ED to engage in ACP conversations, modeled from previously successful ED-based behavioral interventions.^15–22^ The intervention consists of establishing rapport with the patients, assessing their readiness to discuss ACP, and setting up the next steps of discussing ACP with their providers.^21–23^ A pilot study among 50 seriously ill older adults revealed that 83% of patients found the physician-led intervention acceptable and that it may have motivated them to engage in ACP conversations after leaving the ED.^21,22^ Yet physicians were often interrupted when they were conducting the pilot intervention^22^ and during regular clinical care,^24–26^ limiting its potential efficacy. At the same time, multiple nurses who witnessed *ED GOAL* with their patients expressed interest in performing *ED GOAL*.

Nurse-led ACP interventions have been shown to reduce patients’ decisional conflict and increase the documentation of care preferences.^27^ An international expert panel recommends the initiation of ACP by nonphysicians.^28^ Nurses may be well positioned to initiate ACP because they have a unique strength in communication skills,^29^ spend more time at the bedside, and perform motivational interviews as part of their scope of practice.^30,31^ Despite the theoretical advantages demonstrated in other clinical care settings, the empiric evidence for a nurse-led ACP intervention in the ED is lacking. Therefore, we sought to determine full-time clinical staff nurses’ perceptions regarding facilitating ACP conversations in the ED.

## Methods

### Study design and setting

We conducted a survey to examine full-time ED nurses’ perceptions of facilitating ACP conversations in general in the ED at one academic and one community hospitals. We chose to only include full-time nurses in our study because we are hoping to identify ACP nurse champions within our department for future studies. Both EDs are located within the northeastern region of the United States with a total volume of 70,000 visits annually. This study was approved by the Partners Institutional Review Board. We followed the STROBE criteria for reporting of our results.^32^

### Participants and procedure

We included nurses who worked primarily in the ED setting at one of the sites. We excluded participants who identified as float or part-time nurses working <32 hours per week. Potential participants were identified by nursing directors. We requested that nursing directors of both EDs send out study recruitment e-mails with an electronic survey link of the study to the sites’ ED nursing e-mail distribution list. In addition, trained research assistants approached nurses to complete the survey in person, either online or on paper, over four weeks. Study data were collected using Research Electronic Data Capture (REDCap), a secure web-based software platform designed to support data capture for research studies.^33,34^

### Survey development

Because validated surveys to measure nurses’ perception of facilitating ACP conversations in the ED do not exist, a panel of experts with expertise in ED nursing care (B.B., N.A.E., and K.O.), nursing research (T.F.G. and D.L.B.), and ACP conversations in the ED (K.O.) designed the survey. The survey was iteratively refined in content and language used until the study team found it clinically relevant and acceptable for administration.

To measure nurses’ perceptions of facilitating ACP conversations, we used a 5-point Likert-type scale (1 = not at all, 2 = slightly, 3 = somewhat, 4 = very, 5 = extremely) and a series of multiple-choice questions. In addition to nurses’ perceptions, we also assessed the prevalence of patients who would potentially benefit from ACP conversations in the ED and explored participants’ interest and comfort level in facilitating such conversations. Their perceptions of potential models for training nurses to assist patients with ACP conversations were also explored. We also collected demographic information of the nurse participants, including gender, age range, clinical experience, clinical certifications, and union membership status.

### Statistical methods

For descriptive statistics, data were described as frequency and percentage. Mann–Whitney *U* test was used to compare the means of groups of nurses by gender and hospital sites (academic and community EDs), whereas Kruskal–Wallis *H* test was used for age groups and total clinical experience. Analyses were performed using IBM SPSS Version 24.^35^

## Results

We identified 145 full-time (>32 work hours per week) nurses. Among the eligible nurses, 77 completed the survey (53.1% response rate). The reliability of the survey responses demonstrated the Cronbach’s α of 0.72. The study participants were primarily female (61, 79.2%), more than half were between 31 and 55 years of age (43, 55.8%), and 57 (74.0%) worked at the academic ED. Forty-five participants (58.4%) reported having had at least 10 years of total clinical experience. Most nurses had a Bachelor of Science in Nursing (BSN) (65, 84.4%), whereas one (1.3%) had a doctorate. Twenty-two (28.6%) had Certified Emergency Nurse (CEN) certification, and two were Sexual Assault Nurse Examiner (SANE) certified. All (98.7%) but one participant were members of a nursing union (Table 1).

When asked about the frequency of encountering patients who would benefit from ACP conversations, most participants (51, 66.2%) responded that they encountered between 3 and 5 patients in a typical shift, whereas 12 participants (12, 15.6%) encountered 6 or more patients. All participants perceived the conversation as at least somewhat important to be discussed in the ED. Most nurses expressed an interest in taking an active role (67, 87.0%) to help patients clarify their end-of-life care preferences and to receive training to improve their communication skills in this area (61, 79.2%).

**FIG. 1.**
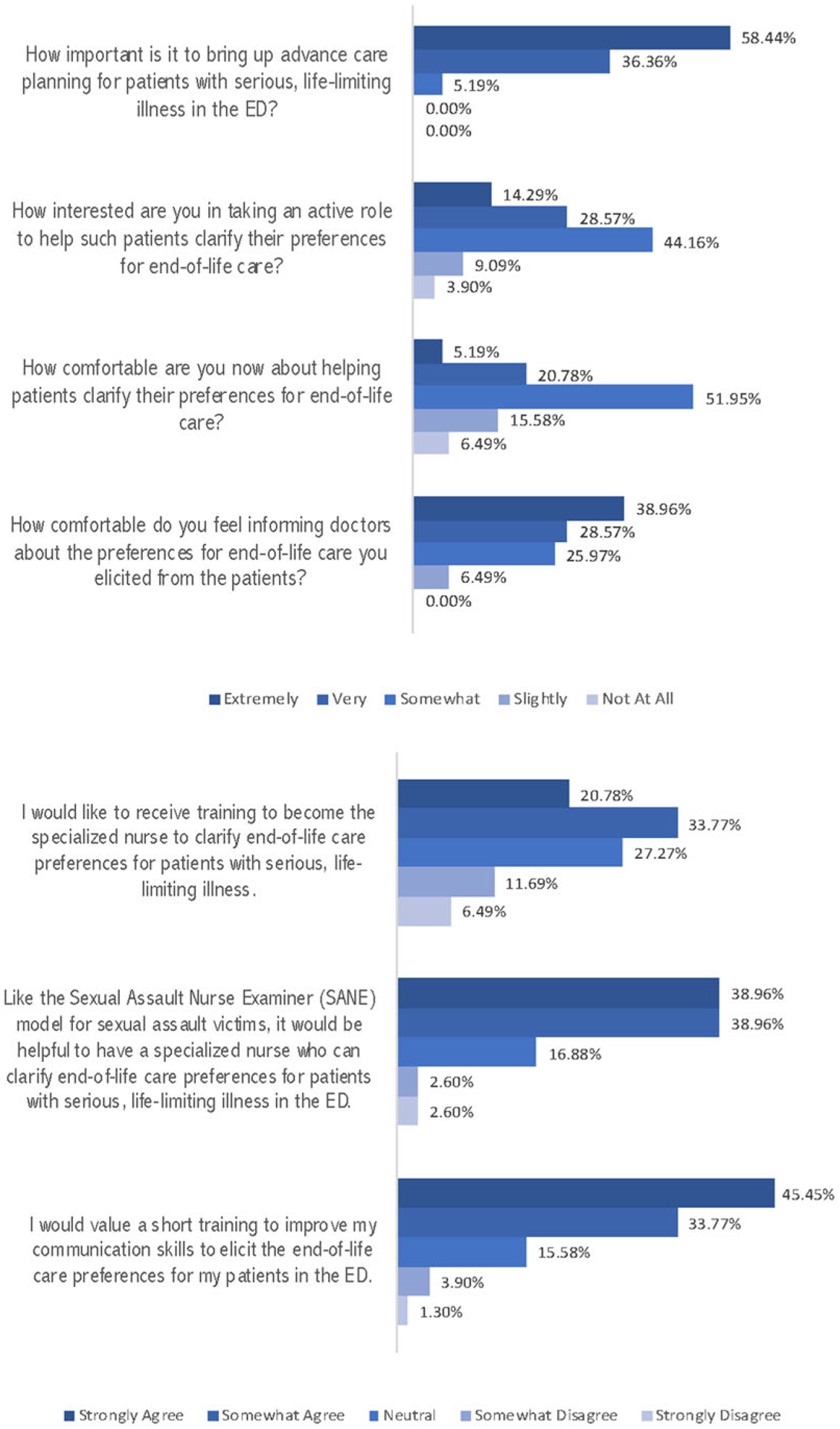
Participant’s responses on advance care planning in the ED. ED, emergency department.

Forty nurses (51.9%) felt somewhat comfortable in helping patients clarify their preferences for end-of-life care. The majority of the participants (60, 77.9%) agreed that a specialized nurse model, akin to the existing SANE, would be helpful. All felt comfortable in informing physicians about the end-of-life preferences they obtained from patients (Fig. 1).

No significant differences were found when comparing the perceived interest and comfort in facilitating ACP conversations between the two sites in our studies, genders, and different age groups. Nurses’ interest to facilitate ACP conversations is associated with the different levels of total clinical experience *χ*^2^ (2) = 6.2, *p* = 0.045, with a mean rank interest score of 39.5 for nurses with <5 years, 28.8 between 5 and 10 years, and 43.1 with >10 years of total experience.

## Discussion

Our findings suggest that ED nurses considered ACP conversations as at least “somewhat important” and were willing to play an active role in initiating such conversations. The respondents also recommended training to improve their communication skills to initiate ACP conversations. Early (<5 years) and later career nurses (>10 years) were more likely to be interested in facilitating ACP conversations than those between 5 and 10 years experience. We hypothesized that nurses who had between 5 and 10 years of experience might have more responsibilities at work and thus were less interested in participating in a new undertaking. The majority of the participants suggested a specially trained nurse consultation model would help conduct these difficult conversations in the ED.

National models for specially trained consultation nurses exist in the ED. Venous access nurses (i.e., nurses who insert ultrasound-guided intravenous access) and SANE are the most common examples. The SANE model has been shown to result in a higher quality of care than any other clinician in the ED for specific types of care.^36–38^ A specially trained nurse consultation model may be more practical than physicians-led or social workers-led models (social workers are often unavailable in most EDs). Furthermore, alternative training models in end-of-life care (e.g., End-of-Life Nursing Education Consortium [ELNEC] curriculum and hospice and palliative care nurses certification^39,40^) may also be helpful. A study that looked at a nurse-led ACP program has been shown to lessen patients’ decisional conflict as well as increase the documentation of care preferences (e.g., advance directives and do not resuscitate orders in other clinical settings).^27^ Based on our study results, a nurse-led ACP intervention implemented as a specially trained nurse consultation model may be worth exploring. Future research should explore the feasibility of training a subgroup of ED nurses to become the specially trained nurses for ACP. If successful, the specialized board certification process based on competency with a train-the-trainer model could be established, similar to the SANE model.^41^

Our study has several limitations. The participants were recruited at one academic and one community hospital, which may have influenced their responses based on their clinical experience. The patient demographics that created the clinical experience at these hospitals were likely similar to other institutions in the United States. Participation bias captured more responses from nurses interested in ACP conversations. The responses are likely internally valid, given that we were primarily interested in the perceptions of such interested nurses in the ED. We are aware that only a subset of nurses may be interested in becoming specially trained in ACP conversations. The principal investigator is an attending emergency physician, so it is possible that the ED nurses’ responses, though deidentified, might be biased toward a more positive outlook.

## Conclusion

ED nurses perceived ACP conversations as important and felt comfortable in facilitating them in the ED. Specially trained nurses may play a pivotal role in initiating these conversations with patients receiving care in the ED.

## Figures and Tables

**Table 1.** Participants’ Characteristics

Participants	Results (*N* = 77), *n* (%)
Demographics	
Female	61 (79.2)
Age, years	
<31	18 (23.4)
30–55	43 (55.8)
>55	16 (20.8)
Highest education	
ADN	7 (9.1)
BSN	65 (84.4)
Master’s	4 (5.2)
DNP	1 (1.3)
Clinical experience	
Total, years	
<5	13 (16.9)
5–10	19 (24.7)
>10	45 (58.4)
ED	
<5	21 (27.3)
5–10	18 (23.4)
>10	38 (49.4)
Nursing union	
Yes	75 (98.7)

ADN, Associate Degree in Nursing; BSN, Bachelor of Science in Nursing; DNP, Doctor of Nursing Practice; ED, emergency department.

## Abbreviations Used

ACP: advance care planning
ADN: Associate Degree in Nursing
BSN: Bachelor of Science in Nursing
DNP: Doctor of Nursing Practice
ED: emergency department
SANE: Sexual Assault Nurse Examiner
